# Predictive factors of prognosis after radiation and steroid pulse therapy in thyroid eye disease

**DOI:** 10.1038/s41598-019-38640-5

**Published:** 2019-02-14

**Authors:** Makoto Ito, Yasuhiro Takahashi, Eisuke Katsuda, Yukihiko Oshima, Arisa Takeuchi, Toshie Mori, Souichirou Abe, Yoshimasa Mori, Hirohiko Kakizaki, Kojiro Suzuki

**Affiliations:** 1 Department of Radiology, Aichi Medical University Hospital, Aichi, Japan; 2 Department of Oculoplastic, Orbital, and Lacrimal Surgery, Aichi Medical University Hospital, Aichi, Japan

## Abstract

To identify predictive factors of prognosis after radiotherapy with concurrent steroid pulse therapy for thyroid eye disease, retrospective analyses were performed among 77 patients. Clinical activity score and magnetic resonance imaging were used to evaluate degrees of orbital inflammation. As a pre-treatment work-up, the thyroid-stimulating antibody (TSAb) level was measured. During a median follow-up of 25.0 months, the 2-year cumulative relapse-free rate (CRFR) was 80.9%. In the univariate analysis, a worse 2-year CRFR was significantly associated with the presence of optic neuropathy (*P* = 0.001), a higher TSAb rate (*P* = 0.001), and lower standard deviation (SD) of signal intensity at the extraocular muscle in T2-weighted images (*P* = 0.006). In the multivariate analysis, TSAb rate and SD affected the CRFR independently. When TSAb activity of 2800% was set as a cut-off at 2 years after treatment, the predictive sensitivity and specificity of relapse were 81.2% and 90.6%, respectively. With regard to SD, the respective sensitivity and specificity values were 81.2% and 82.7% when 100 was set as a cut-off. In conclusion, high TSAb and low SD were significant risk factors for cumulative relapse in orbital radiotherapy. Cut-off values of 2800% for TSAb and 100 for SD may be suitable.

## Introduction

Thyroid eye disease (TED) is a periorbital autoimmune disease that disfigures the face and reduces visual function. The majority of patients with TED do not require intensive treatment^1^ because their symptoms remain mild or resolve spontaneously. However, 5–30% of patients experience moderate to severe symptoms such as lagophthalmos and subsequent corneal impairment due to proptosis, eyelid retraction, double vision, and optic nerve compression that require treatment^2^.

The adequate management of TED requires ongoing assessment of disease activity. Clinical activity score (CAS) is widely used for the evaluation of TED activity^3^, however, magnetic resonance imaging (MRI) may be more reliable for the objective evaluation of orbital inflammation in TED^4,5^. Combined radiotherapy and steroid pulse therapy is evidently more effective for reduction of TED-related orbital inflammation than either treatment alone, with approximately 80% of cases exhibiting favourable reduced inflammation^1,6,7^, though orbital inflammation does sometimes relapse. Understanding predictive factors pertaining to TED prognosis may be helpful with regard to determining the need for frequent follow-up and more aggressive treatment. However, the relevant factors after radiotherapy and concurrent steroid pulse therapy remain unclear. The aim of the current retrospective study was to investigate predictive factors for TED prognosis after radiotherapy and steroid pulse therapy.

## Results

Patient characteristics are summarised in Table 1. To maintain a euthyroid state, 66 patients were treated with antithyroid agents, seven with surgery and two with radioactive iodine; however, at the time of treatment, 13 patients were in a hyperthyroid state and 8 were in a hypothyroid state. Thirty-four patients underwent some type of treatment for TED before the combination therapy, therefore, 16 patients with more than 24 months duration of ophthalmopathy were included. All patients completed the prescribed treatment without any long delays, and the median follow-up period was 25.0 (range 6.2–106.1) months.

**Table 1:** Patient characteristics.

All cases (N = 77)		
Age (years)	Median	58
	Range	(25–80)
Male:female		29:48
Duration of ophthalmopathy (months)	Median	7.3
	Range	1.1–43.2
Thyroid function at radiotherapy	Hyperthyroid	13
	Euthyroid	56
	Hypothyroid	8
Previous treatment for hyperthyroidism	Anti-thyroid agent	66
	Radioactive iodine	2
	Surgery	7
	None	9
Previous treatment for ophthalmopathy	Local corticosteroids	4
	Systemic corticosteroids	33
	Surgery	6
	None	43
Number of smokers		24
Number of DM		3
Total dose of mPSL (mg)	Median	5625
	Range	(2250–9000)
CAS at radiotherapy	2–3	37
	4–5	30
	6–7	10
TSAb (%)	Median	1282.4
	Range	(166.0–8045.0)
SD of signal intensity in the ROI	Median	113.5
	Range	(25.9–268.2)
Follow-up time (months)	Median	25.0
	Range	(6.2–106.1)

DM, diabetes mellitus; mPSL, methylprednisolone; CAS, clinical activity score; TSAb, thyroid stimulating antibody; SD, standard deviation; ROI, region of interest.

Data on changes in the parameters investigated after treatment are shown in Table 2. The rate of cases with responders at the initial evaluation after treatment (median 3.3 months, range, 1.9–5.8 months) was 79.2%, and 20.8% were classified as no change. No patients were classified as having progressive disease. Extraocular muscle thickness, signal intensity ratio (SIR), proptosis, and CAS improved significantly after treatment (all *P* < 0.001). The 2-year cumulative relapse-free rate (CRFR) was 80.9% (95% confidence interval [CI] 69.1–88.5%). Rehabilitative oculoplastic surgery was performed in 18 patients after confirmation of settlement of orbital inflammation. Relapses were observed 2.3 to 47.2 months (median 10.2 months) after the completion of radiotherapy in 17 patients. Of these 17 patients, 6 underwent additional steroid pulse therapy, 4 underwent orbital decompression, 4 underwent both, and the remaining 3 declined any further treatment. No patients underwent re-irradiation.

**Table 2:** Changes in parameters at initial treatment evaluation.

	Pre-treatment	Initial treatment evaluation	*P*
	mean ± SD/median (range)	mean ± SD/median (range)	
Area of ROI	69.7 cm^2^ ± 25.4	53.8 cm^2^ ± 19.8	<0.001
SIR	1.72 ± 0.47	1.28 ± 0.34	<0.001
Ocular proptosis	21.2 cm ± 2.64	19.9 cm ± 2.58	<0.001
CAS	4 (2–7)	1 (0–5)	<0.001

SD, standard deviation; ROI, region of interest; SIR, signal intensity ratio; CAS, clinical activity score.

**Figure 1:**
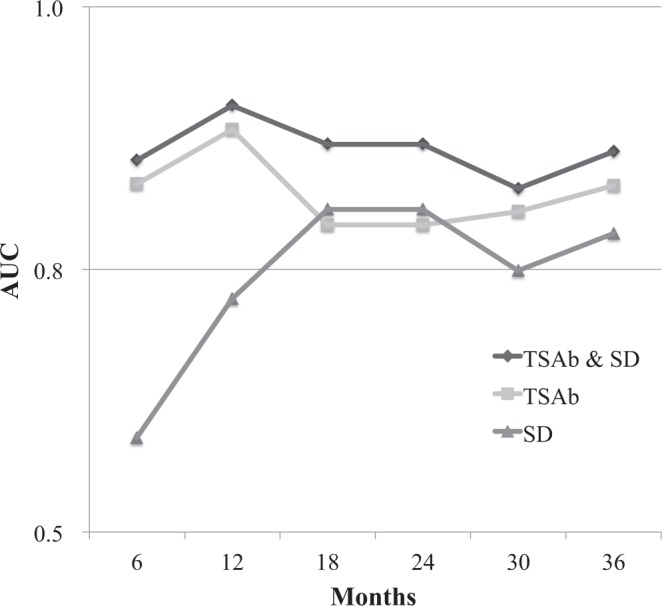
Chronological results of ROC analysis. The relationships between relapse and prediction factors were analysed. TSAb had a higher AUC than SD, especially in the early period. A novel parameter derived by combining TSAb and SD had a higher AUC than either parameter alone. AUC, area under the curve; ROC, receiver operating characteristic; SD, standard deviation; TSAb, thyroid stimulating antibody.

The results of Fisher’s exact test performed on initial response data and univariate analysis performed on CRFR data are shown in Table 3. Only patients with higher thyroid-stimulating antibody (TSAb) rates had significantly worse initial responses (P < 0.05). In univariate analysis, a worse 2-year CRFR was significantly associated with the presence of optic neuropathy (47.7% vs. 86.3%, *P* = 0.001), higher TSAb rates (66.5% vs. 93.1%, *P* = 0.001), and lower region of interest (ROI) standard deviations (SDs) (67.7% vs. 94.1%, *P* = 0.006). In multivariate analysis, the TSAb rate (hazard ratio 1.010, 95% confidence interval [CI] 1.004–1.014, *P* < 0.001) and SD (hazard ratio 0.974, 95% CI 0.957–0.980, *P* < 0.001) affected the CRFR independently. Based on the results of multivariate analysis, additional analyses pertaining to the relationships between relapse and TSAb and SD were performed. When TSAb activity of 2800% was set as a cut-off point, predictive sensitivity and specificity of relapse were 81.2% (95% CI 54.4–96.0%) and 90.6% (95% CI 79.7–96.9%), respectively. When an SD of 100 was set as a cut-off point, sensitivity was 81.2% (95% CI 54.4–96.0%) and specificity was 82.7% (95% CI 69.7–91.8%). Chronological results of ROC analysis of the relationship between relapse and predictive factors are shown in Fig. 1. Respective AUCs for TSAb rate, SD, and a parameter derived from a combination of TSAb rate and SD were 0.83, 0.59, and 0.86 at 6 months, 0.88, 0.72, and 0.91 at 12 months, and 0.79, 0.80, and 0.87 at 24 months.

**Table 3:** Fisher’s exact test analysing initial response and univariate CRFR analysis.

		Responders			2-year CRFR (%)	*P*
		Yes	No	*P*		
Age (years)	<58 (*n* = 40)	31	9	0.783	81.4	0.683
	≥58 (*n* = 37)	30	7		79.7	
Sex	Male (*n* = 29)	26	3	0.0915	91.8	0.131
	Female (*n* = 48)	35	13		74.5	
Duration of ophthalmopathy (months)	<7.3 (*n* = 39)	31	8	0.99	80.1	0.899
	≥7.3 (*n* = 38)	30	8		82.2	
Thyroid function at radiotherapy	Euthyroid (*n* = 56)	43	13	0.534	82.9	0.851
	Dysfunction (*n* = 21)	18	3		74.7	
Previous treatment for hyperthyroidism	Yes (*n* = 68)	56	12	0.0832	83.4	0.176
	No (*n* = 9)	5	4		59.3	
Previous treatment for ophthalmopathy	Yes (*n* = 34)	27	7	0.99	82.2	0.65
	No (*n* = 43)	34	9		79.4	
Smoker	Yes (*n* = 24)	18	6	0.556	77.8	0.692
	No (*n* = 53)	43	10		82.5	
DM	Yes (*n* = 3)	2	1	0.51	66.7	0.615
	No (*n* = 74)	59	15		81.5	
Optic neuropathy	Yes (*n* = 11)	8	3	0.689	47.7	0.001
	No (*n* = 66)	53	13		86.3	
Total dose of mPSL (mg)	<5625 (*n* = 36)	29	7	0.99	86.1	0.171
	≥5625 (*n* = 41)	32	9		76.0	
Post oral administration of mPSL	Yes (*n* = 22)	17	5	0.765	67.9	0.094
	No (*n* = 55)	44	11		86.0	
CAS	<4 (*n* = 37)	26	11	0.091	76.9	0.461
	≥4 (*n* = 40)	35	5		83.8	
SIR	<1.64 (*n* = 35)	27	8	0.99	82.9	0.283
	≥1.64 (*n* = 33)	25	8		78.2	
TSAb	<1282.4 (*n* = 35)	32	3	0.034	93.1	0.001
	≥1282.4 (*n* = 34)	24	10		66.5	
SD	<113.5 (*n* = 34)	25	9	0.776	67.7	0.006
	≥113.5 (*n* = 34)	27	7		94.1	

CRFR, cumulative relapse-free rate; DM, diabetes mellitus; mPSL, methylprednisolone; CAS, clinical activity score; SIR, signal intensity ratio; TSAb, thyroid stimulating antibody; SD, standard deviation.

Twenty-two adverse events requiring medical intervention were observed in a total of 19 patients. These adverse events consisted of deterioration of dry eye in 13 cases, hepatitis in 3 cases, diabetes in 2 cases, cataract in 3 cases, and gastric ulcer in 1 case. All of these events were mild or moderate, and none were associated with serious sequelae. There was no significant correlation between the total dose of corticosteroid and the incidence of adverse events. The 3 patients who underwent a cataract operation were aged 58, 61, and 71 years at the time of the operation, and the relevance of irradiation and steroid administration was unclear. Although only 5 cases had been followed up for ≥7 years after treatment, secondary cancer did not develop during the follow-up period in any of these cases.

## Discussion

The present study is the first report of an association between a higher TSAb rate and worse initial response or worse 2-year CRFR after radiotherapy with steroid pulse therapy. The results of the study suggest that a TSAb activity cut-off of 2800% is useful for determining the predictive sensitivity and specificity of relapse. TSAb is an autoantibody that induces orbital inflammation via receptors expressed in orbital tissues, and correlates with the degree of orbital inflammation in TED^8,9^. Accordingly, we suggest that it is reasonable that higher TSAb activity is a factor relevant to a higher relapse rate after radiotherapy with steroid pulse therapy.

We used SDs to objectively and quantitatively evaluate the homogeneity of extraocular muscles via MRI, and found that lower SDs were significantly associated with worse 2-year CRFRs. Lower SDs denote a homogeneous extraocular muscle signal and may indicate more severe fatty infiltration and intramuscular oedema, which may have resulted in the higher relapse rate observed in the current study. Notably, SDs are not suitable for making predictions in cases of early relapse (Fig. 1). Using the marker combined with TSAb is preferable, especially in the early period.

Although TED in the active phase tends to be uniformly treated with an irradiation dose of 20 Gy, we propose a tailor-made treatment strategy whereby the intensity of treatment is based on predictive factors. Some studies have investigated reducing the total doses of irradiation or steroid administration^10,11^. Although it should not be applied to all cases uniformly, reducing the treatment intensity in patients without adverse prognostic factors is worthy of consideration. Conversely, in patients with adverse prognostic factors increases in total steroid or irradiation doses may be appropriate. Notably however, a total steroid dose of >8 g can cause fatal hepatopathy, and in a previous study there was reportedly no benefit associated with dose-escalation in routine orbital radiotherapy^12^. A combination of rituximab, cyclosporine, and somatostatin analogues may reduce the risk of relapse^13^. Further studies are needed to establish better treatment strategies for cases with and without adverse prognostic factors.

In a previous pilot study including 25 patients with moderately severe TED, pre-treatment CAS was significantly higher in patients who responded to radiotherapy (3.7 vs. 2.1; *P* = 0.008)^14^. Orbital inflammation was not evaluated via MRI in that study, however, mean pre-treatment CAS in the non-responders was 2.1, raising doubt as to whether all participants actually had orbital inflammation. In the current study, orbital inflammation activity was evaluated via CAS and MRI, and patients were deemed to be in active phase prior to radiotherapy with concurrent pulse therapy.

A previous study measured the SIR of enlarged extraocular muscles and cerebral substantia alba at the coronal section in STIR images from patients with TED who were treated with steroid pulse therapy^15,16^. The results of that study were inconsistent with the observation in the current study that SIR was significantly greater in patients in which treatment was effective than it was in those in which treatment was ineffective. A possible reason for this discrepancy is that different measurement parameters were used in the two studies, such as the position of the ROI, the size of the ROI, and the setting of pulse sequences.

In a previous MRI study, more favourable outcomes (reduction in muscle size or recovery from diplopia) were reported after combined treatment in patients with uniform T2-weighted image (T2WI) intermuscular intensity than in those with a non-uniform pattern^16^. There was no significant relationship between the SD and the effectiveness of combined therapy in the current study, but this difference may be due to the difference in the methods used to determine therapeutic effect in the two studies.

The current study had several limitations. Because the study design was retrospective, 16 patients were administered higher or lower methylprednisolone for pulse therapy. Furthermore, 22 patients were prescribed subsequent oral prednisolone, 34 patients underwent previous treatment for ophthalmopathy, and duration of ophthalmopathy had a large range (1.1–43.2 months). These limitations were mainly due to the selection criteria; patients who had a treatment history (in another hospital) were included in our study group. This introduced the possibility of confounding variables in the patient characteristics. Although the effectiveness of the treatment was evaluated 3 months post-treatment in most patients, some patients were evaluated at earlier or later follow-up time-points (median 3.3 months, range, 1.9–5.8 months).

In conclusion, high TSAb values and low SD values were significant risk factors for cumulative relapse after orbital radiotherapy with concurrent steroid pulse therapy for TED. Furthermore, a novel parameter derived by combining TSAb and SD was a more sensitive prognostic predictor than either parameter alone. We propose the utilisation of a tailor-made treatment strategy in patients with and without prognostic predictive indicators.

## Methods

### Ethics approval and study design

The institutional review board of Aichi Medical University Hospital, Japan, approved this retrospective observational study (application number 2017-H211). The study was conducted in accordance with the tenets of the Declaration of Helsinki and its subsequent amendments. Written informed consent was obtained from all patients.

### Patients

Patients with TED who were diagnosed in the active phase using CAS and/or MRI underwent radiotherapy with steroid pulse therapy at the Aichi Medical University Hospital. Data pertaining to 115 consecutive patients who underwent radiotherapy for active TED between August 2005 and March 2017 were initially reviewed. A total of 38 were subsequently excluded: 19 due to low CAS (0–1), 8 because they did not undergo steroid pulse therapy, 6 due to a short follow-up period (less than 6 months), and 5 due to insufficient ophthalmic examination data availability. The remaining 77 patients were included in the study.

### Laboratory tests and ophthalmic evaluations

Blood tests for pre-treatment work-up included a complete blood count; assessment of thyroid function, TSAb activity, liver and kidney functions; and hepatitis B and C serologic analyses. The TSAb measurement method at our institution was changed from the radio-immuno-assay (RIA) to the enzyme-immuno-assay (EIA) on 1 July 2014. Therefore, for a meaningful comparison, RIA-TSAb values were converted to the corresponding EIA-TSAb values via the following formula^17^:

1 $${\rm{EIA}} \mbox{-} {\rm{TSAb}}\,{\rm{value}}=(0.998\times {\rm{RIA}} \mbox{-} {\rm{TSAb}}\,{\rm{value}})+142.7$$

Ophthalmologic examinations, performed by expert ophthalmologists before treatment intervention, included visual acuity, intraocular pressure, presence or absence of lid lag, measurement of eyelid position (margin reflex distances 1 and 2), Hertel exophthalmometric measurement, CAS, extraocular muscle motility evaluation using the Hess chart and binocular single vision field, and slit-lamp and funduscopic examinations.

### Steroid therapy

Most patients (n = 61) underwent two or three cycles of steroid pulse therapy under a regimen of body weight ×10 mg/kg/day of methylprednisolone for 3 days per cycle. Patients treated early in the study period (n = 13) were administered 1000 mg of methylprednisolone per day, and 3 patients were administered reduced doses. Patients did not receive subsequent oral prednisolone in principle. However, in order to avoid sudden discontinuation, oral prednisolone (initial dose 0.2–0.4 mg/kg per day, tapering and discontinued 1–3 months after initial administration) was administered to 22 patients who already had been prescribed in a previous hospital.

### Radiotherapy

Both the target area and orbital structures for which irradiation was avoided were contoured using treatment planning systems (either XiO, Electa, CMS, St Louis, MO, USA or Eclipse, Varian Medical System, Palo Alto, CA, USA). The clinical target volume was defined utilising the extraocular muscles and retrobulbar soft tissue, and the planning target volume was defined as the clinical target volume with an additional 0.5-cm margin. Irradiation was delivered to the planning target volume using 6-MV photons, with lateral opposing fields angled posteriorly at 2–4° to align the anterior field edges. Although a 0.5-cm multi-leaf collimator margin was utilised, the anterior-posterior margin was reduced to spare the lens and pituitary gland. A total dose of 20 Gy in 10 fractions was delivered to all but three patients during hospitalisation for steroid pulse therapy. Due to their young age, two patients received 12 Gy in six fractions, and one patient received 10 Gy in five fractions.

### MRI examination

Axial and coronal orbital MR images were obtained via a 1.5-Tesla scanner (MAGNETOM Avanto, Siemens Health-care, Erlangen, Germany) with a head surface coil. Typical pulse sequence parameters were spin-echo 4000/100 (repetition time ms/echo time ms) for T2WIs and inversion recovery 4000/180/80 (repetition time ms/inversion time ms/echo time ms) for short-tau inversion recovery (STIR) images. Section thickness, field of view, and matrix were 3–5 mm, 140 × 140 mm, and 256 × 220, respectively. Extraocular muscle thickness, proptosis, and SIR were measured using the digital calliper tool of a viewer (ShadeQuest/ViewR, Yokogawa Medical Solutions Corporation, Tokyo, Japan).

**Figure 2:**
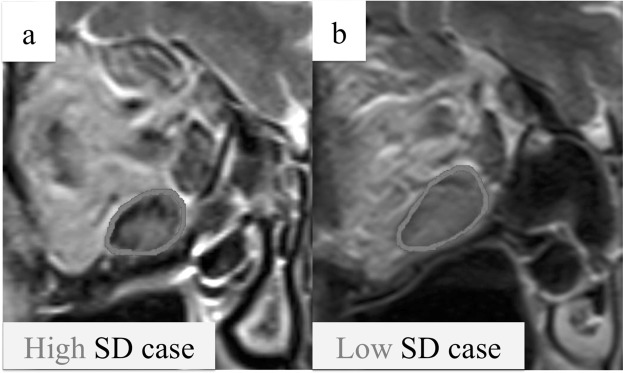
Definition of ROI and representative cases of high and low SD. The ROI was placed along the perimeter of the greatest enlarged extraocular muscle in a coronal section T2WI, and the SD of the inside signal intensity was measured. (**a**) A representative case of high SD. (**b**) A representative case of low SD. ROI, region of interest; SD, standard deviation; T2WI, T2-weighted image.

**Figure 3:**
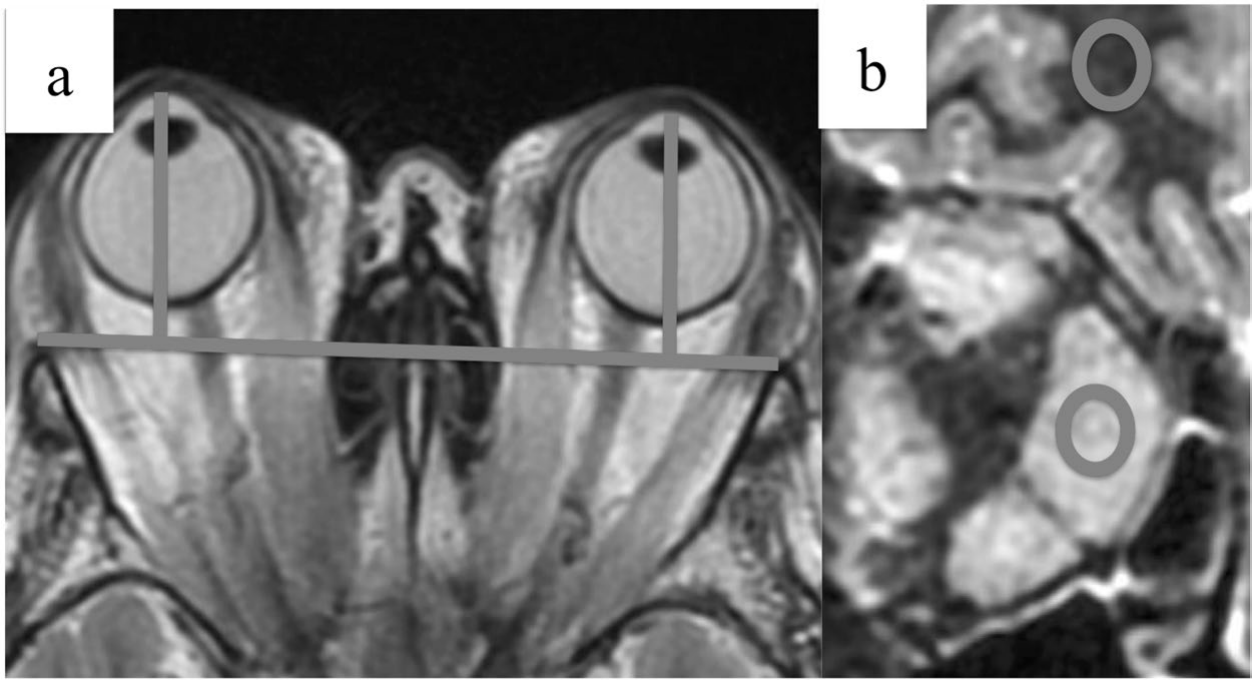
Measurement of proptosis and SIR. (**a**) Proptosis was defined as the distance between the corneal eminence and the connecting line of both zygomatic bones in an axial section T2WI. (**b**) SIR was defined as the ratio of enlarged muscular signal intensity to cerebral white matter in the STIR image. SIR, signal intensity ratio; STIR, short-tau inversion recovery; T2WI, T2-weighted image.

Referring to the previous study, the following parameters were measured by expert radiologists^15,16^. Extraocular muscle thickness was defined as the area of the region of interest (ROI), which was situated along the perimeter of the largest cross-section of the extraocular muscles in the coronal T2WI (Fig. 2). In conjunction with placing the ROI, the SD of the internal signal intensity was measured. Representative cases with high and low SDs are shown in Fig. 2a,b, respectively. Axial globe position was defined as the distance between the corneal eminence and the interzygomatic line on an axial T2WI through the optic nerve (Fig. 3a). The SIR was defined as the ratio of signal intensity of the muscles to that of the cerebral white matter (Fig. 3b).

### Data collection and statistical analyses

Initial treatment response was evaluated in all cases based on the 10-point CAS at pre- and post-treatment. ‘Responders’ were defined as patients for who the CAS decreased by 2 or more points. A change in CAS of 0 or 1 was classified as ‘no change’, and ‘progressive disease’ was assigned to patients for who the CAS increased by 2 or more points. During the follow-up period, cases requiring additional salvage anti-inflammatory therapy (including cases in which the patient refused treatment) were defined as cases of relapse. CRFR was calculated from the initial date of treatment until the last follow-up or the date of relapse. The association of characteristics with initial response was analysed using Fisher’s exact test for categorical variables and for continuous variables. Student’s unpaired *t*-test was used to determine the significance of differences between two sample means. Because CAS was an ordinal variable, the Wilcoxon signed-rank test was used to compare it before and after treatment. CRFR was estimated using the Kaplan-Meier method. Log-rank tests were used to compare the estimated CRFR among the subgroups in univariate analysis. The Cox proportional hazards model was used in multivariate analysis. *P* < 0.05 was considered statistically significant. Factors yielding a significance of *P* < 0.1 in univariate analysis were included in the multivariate analysis. The receiver operating characteristic (ROC) curve was used to evaluate relationships between relapse and selected variables. Based on the Youden index, the cut-off point was determined and well-balanced sensitivity and specificity values were obtained^18^. The predictive capacities of each of the selected variables were compared via area under the curve (AUC) analysis. All statistical analyses were performed with EZR version 1.33 (Saitama Medical Center, Jichi Medical University, Saitama, Japan), which is based on the R and R commander^19^.

## Data Availability

The datasets generated during and/or analysed during the current study are available from the corresponding author on reasonable request.
